# Artificial Intelligence-Powered Quality Assurance: Transforming Diagnostics, Surgery, and Patient Care—Innovations, Limitations, and Future Directions

**DOI:** 10.3390/life15040654

**Published:** 2025-04-16

**Authors:** Yoojin Shin, Mingyu Lee, Yoonji Lee, Kyuri Kim, Taejung Kim

**Affiliations:** 1 College of Medicine, The Catholic University of Korea, 222 Banpo-Daero, Seocho-gu, Seoul 06591, Republic of Korea; yoojins07@catholic.ac.kr (Y.S.); mkl0107@catholic.ac.kr (M.L.); gina228@catholic.ac.kr (Y.L.); 2 College of Medicine, Ewha Womans University, 25 Magokdong-ro 2-gil, Gangseo-gu, Seoul 07804, Republic of Korea; kkyuri01@ewhain.net; 3 Department of Hospital Pathology, Yeouido St. Mary’s Hospital, College of Medicine, The Catholic University of Korea, 10, 63-ro, Yeongdeungpo-gu, Seoul 07345, Republic of Korea

**Keywords:** artificial intelligence, neural network, quality control, image analysis, tissue classification, prognosis prediction

## Abstract

Artificial intelligence is rapidly transforming quality assurance in healthcare, driving advancements in diagnostics, surgery, and patient care. This review presents a comprehensive analysis of artificial intelligence integration—particularly convolutional and recurrent neural networks—across key clinical domains, significantly enhancing diagnostic accuracy, surgical performance, and pathology evaluation. Artificial intelligence-based approaches have demonstrated clear superiority over conventional methods: convolutional neural networks achieved 91.56% accuracy in scanner fault detection, surpassing manual inspections; endoscopic lesion detection sensitivity rose from 2.3% to 6.1% with artificial intelligence assistance; and gastric cancer invasion depth classification reached 89.16% accuracy, outperforming human endoscopists by 17.25%. In pathology, artificial intelligence achieved 93.2% accuracy in identifying out-of-focus regions and an F1 score of 0.94 in lymphocyte quantification, promoting faster and more reliable diagnostics. Similarly, artificial intelligence improved surgical workflow recognition with over 81% accuracy and exceeded 95% accuracy in skill assessment classification. Beyond traditional diagnostics and surgical support, AI-powered wearable sensors, drug delivery systems, and biointegrated devices are advancing personalized treatment by optimizing physiological monitoring, automating care protocols, and enhancing therapeutic precision. Despite these achievements, challenges remain in areas such as data standardization, ethical governance, and model generalizability. Overall, the findings underscore artificial intelligence’s potential to outperform traditional techniques across multiple parameters, emphasizing the need for continued development, rigorous clinical validation, and interdisciplinary collaboration to fully realize its role in precision medicine and patient safety.

## 1. Introduction

The landscape of healthcare is undergoing a profound transformation, driven by the convergence of precision medicine and artificial intelligence (AI). Precision medicine has made significant strides through the development of large-scale biological databases (e.g., the human genome sequence), advanced patient characterization methods (e.g., diverse cellular assays), and sophisticated computational tools for analyzing complex datasets [1]. This approach promises to revolutionize healthcare by offering personalized interventions based on a patient’s unique genetic, environmental, and lifestyle factors. However, despite its promise, the integration of precision medicine into clinical practice remains challenging. Physicians face the dual burden of interpreting increasingly complex datasets while managing demanding schedules and administrative tasks, such as electronic health record (EHR) documentation. These pressures contribute to physician burnout, potentially leading to medical errors, reduced productivity, and diminished patient satisfaction [2,3,4,5]. Moreover, despite extensive training and experience, diagnostic and treatment decisions often remain subjective. Variability in factors such as visual perception and data integration among physicians can lead to inconsistencies in patient care [6].

AI has emerged as a powerful tool to address these issues by automating repetitive tasks, improving diagnostic precision, and ensuring consistent quality control across specialties. Broadly defined, AI refers to the capacity of machines to replicate human intelligence—interpreting language, recognizing images, solving problems, and learning from data [7]. Its applications span multiple disciplines, including radiology, surgery, and pathology. Furthermore, AI-powered wearable sensors, drug delivery systems, and biointegrated devices enable highly personalized treatments by optimizing physiological monitoring and therapeutic interventions with precision. For instance, AI algorithms enhance image analysis in diagnostics, assist with surgical workflow recognition, and improve tissue classification and prognosis prediction in pathology. This paper presents a comprehensive review of AI’s role in advancing healthcare quality assurance, examining its applications across these key domains.

What sets AI apart is its ability to process large volumes of complex data with unprecedented speed and accuracy. To overcome the challenges of explicitly coding all aspects of complex tasks, machine learning (ML) automates model building by using data-driven algorithms to detect patterns and generate insights without manual programming. A subset of ML, deep learning (DL), employs neural networks with multiple hidden layers and operations such as convolutions, allowing it to handle high-dimensional and unstructured data more effectively than traditional artificial neural networks [8]. Techniques such as convolutional neural networks (CNNs) for image recognition or recurrent neural networks (RNNs) for sequential data analysis enable AI to outperform conventional approaches in tasks like lesion detection or workflow optimization.

This paper distinguishes itself by offering a holistic analysis of AI’s role in healthcare quality assurance—a domain that has received less attention compared to individual applications like diagnostics or surgery. Unlike most studies that focus narrowly on specific use cases or technical details, this review integrates insights across multiple medical fields while addressing the broader implications for precision medicine. By highlighting both the benefits and challenges of AI adoption, this paper contributes uniquely to advancing the conversation on AI’s transformative potential in healthcare. To provide a comprehensive understanding of the technical aspects, we summarize the types of neural networks and metrics used to assess AI model performance in Table 1 and Table 2, respectively.

## 2. Methodology of This Review

This review was conducted by systematically analyzing studies published between 2015 and 2024 across diagnostic imaging, endoscopy, pathology, and surgery. We focused on peer-reviewed papers reporting AI applications with measurable performance metrics (e.g., accuracy, F1-score, AUROC) compared to conventional methods. The databases searched include PubMed, IEEE Xplore, and Scopus, using keywords such as ‘AI in diagnosis’, ‘CNN in pathology’, and ‘machine learning in surgery’.

## 3. AI in Diagnostics

**Figure 1:**
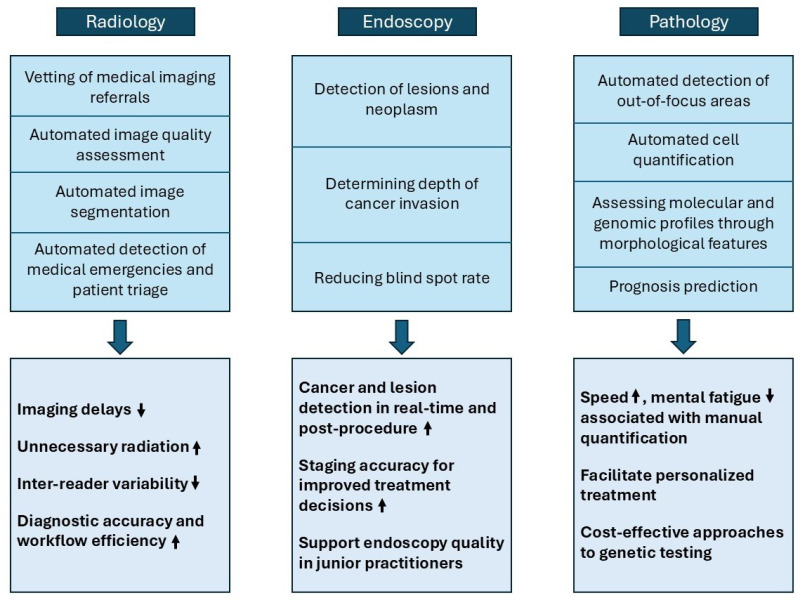
Applications of AI in diagnostic quality control. **Note:** ↑ indicates an increase or improvement; ↓ indicates a decrease or reduction.

AI is profoundly transforming the field of medical diagnostics by enhancing accuracy and efficiency across various specialties. In radiology, AI algorithms are revolutionizing image analysis by enabling precise lesion detection and segmentation, while also automating quality assurance processes to ensure optimal imaging standards. In endoscopy, AI aids in identifying abnormalities with greater sensitivity, improving diagnostic outcomes during procedures. Similarly, pathology has seen significant advancements through AI-driven tools that classify tissues, predict prognosis, and link morphological features to genomic data. These innovations, as illustrated in Figure 1, underscore AI’s potential to streamline workflows and reduce diagnostic variability, ultimately elevating patient care standards.

### 3.1. Radiology

#### 3.1.1. Vetting of Medical Imaging Referrals

Vetting is the process of justifying imaging referrals to prevent unnecessary radiation as well as inappropriate and duplicate examinations. It aims to assess the risks and benefits of medical imaging for patients and determine the suitability of ionizing radiation or alternative imaging methods [15]. Computed tomography (CT) is particularly associated with high radiation doses, and patients undergoing multiple scans face a significantly higher cumulative risk of radiation-induced cancer [16]. Clinically inappropriate or duplicate requests for medical imaging not only impose unnecessary radiation and cost burdens on patients but also add to the workload of radiology departments, leading to imaging delays [17,18]. About 26% of imaging referrals from primary physicians are considered inappropriate, including brain CT for chronic headache, and magnetic resonance imaging (MRI) for acute back pain or osteoarthritis [19]. The percentage of unjustified lumbar spine MRIs can range from 10% to 60% [20], while CT justification rates in Europe show poor performance, ranging between 7% and 30% [16]. Justifying images requires advanced education and training. However, automating the process of referral vetting using machine learning may offer an efficient and cost-effective approach for reducing work burden and imaging delays [18,20].

A study developed both traditional and deep neural models to classify lumbar spine MRI referrals as ‘indicated’ or ‘not indicated’. All models, regardless of type, outperformed eight human radiographers in categorizing 100 referrals [20]. Similarly, a Clinical Decision Support tool called xRefer was used to audit brain CT referrals. xRefer assigns scores on a scale of 0 to 9 based on the ACR scoring system, where 9 indicates a justified referral and 0 indicates an unjustified one. Justification with xRefer demonstrated significantly better consistency between two experts (κ = 0.835) compared to justification without the CDS tool (κ = 0.408) [16].

#### 3.1.2. Automated Image Quality Assessment

Quality assurance (QA) testing is crucial for detecting malfunctions in imaging devices, ensuring they meet established quality standards. However, daily QA may miss subtle scanner faults, potentially leading to poor image quality and misdiagnosis [21]. The ACR QA phantom, for example, has demonstrated limitations in detecting faults within individual MRI coil elements [22].

CNN models have shown promise in identifying scanner faults, achieving an accuracy of 91.56%—surpassing radiographers, who are typically the first line of defense in detecting such issues. This method offers superior accuracy compared to traditional phantom testing and enables real-time monitoring [23]. Additionally, a support vector machine approach can also automate phantom image quality evaluation, addressing subjectivity and repeatability issues with manual assessment [24].

Beyond technical QA, evaluating and rejecting patient images based on quality can also be subjective. While obvious issues, such as cutoff lung fields, are clear grounds for rejection, more subtle factors, like intra-exposure patient motion, may require subjective judgment [25]. CNN models have been developed to assess image quality features, including proper lung inclusion, patient rotation, and inspiration in posterior–anterior chest radiographs. These models achieved an AUROC greater than 0.88 for lung inclusion, and over 0.70 and 0.79 for patient rotation and inspiration, respectively. This approach offers immediate feedback on chest radiograph image quality, helping to minimize delays in diagnosis and reporting [26].

#### 3.1.3. Automated Medical Image Segmentation

Segmentation in medical imaging identifies boundaries of regions of interest (ROIs) in images, crucial for diagnosis, treatment planning, and disease monitoring [27]. Deep learning can automatically identify diseased tissues, eliminating the need for expert-defined segmentations. While segmenting non-diseased organs can be relatively straightforward, determining the extent of diseased tissue is significantly more complex. Current tumor segmentation practices are often limited to basic metrics, such as the largest in-plane diameter, which may not fully capture the tumor’s true size, shape, or heterogeneity [28]. Advances in automated segmentation techniques offer more comprehensive and precise results, enhancing various medical imaging applications. These include quantifying lung opacification in COVID-19 CT scans [29], segmenting brain tumors in MRI scans [30], and measuring total kidney volume in MRI images to monitor disease progression in polycystic kidney disease [31].

#### 3.1.4. Emergency and Trauma Radiology

Appropriate use of emergency imaging has been associated with reduced patient admission rates, shorter hospital stays, and fewer unnecessary surgeries and diagnostic studies [32,33]. On-site or emergency department (ED)-dedicated radiologists interpret imaging results faster, enabling quicker diagnosis and treatment decisions, streamlining patient care, reducing delays, and improving ED workflow efficiency [34]. However, as ED visits and imaging utilization increase, emergency radiology must optimize productivity and efficiency to manage growing demand and resource constraints [35].

Efficient patient triage is crucial in prioritizing the radiologist’s worklist. Machine learning can be employed to automate and enhance the accuracy of acuity scoring systems [36]. The e-triage system uses random forest models to calculate the probabilities of three key outcomes: critical care needs, the requirement for an emergent procedure, and the likelihood of hospitalization. These probabilities are then combined into a single e-triage level, ranging from 1 to 5, with level 1 indicating the highest likelihood of critical care or emergent procedure. This system can adjust to variations in triage protocols and critical care criteria between institutions, such as differences in ICU access. This flexibility allows the system to cater to the specific needs and resources of different EDs, ensuring that high-risk patients receive timely care [37].

AI can also automatically detect medical emergencies with high mortality rates from imaging, significantly improving diagnostic speed and accuracy. Canon’s ^AUTO^Stroke Solution Intracranial Hemorrhage (ICH) algorithm excels in detecting ICH, achieving 93% sensitivity and 93% specificity [38]. A meta-analysis of 40 studies on AI algorithms for detecting ICH in non-contrast CTs/MRIs, along with 19 studies on chronic microbleeds (CMBs) in MRIs, reported overall sensitivity, specificity, and accuracy rates of 92%, 93%, and 93% for ICH detection, and 91%, 93%, and 92% for CMB detection, respectively [39]. AI applications extend to various medical imaging tasks, including the detection of fractures in plain radiographs [40], quantitative visualization of traumatic hemoperitoneum on CT scans [41], ileocolic intussusception in young children on abdominal radiographs [42], and pulmonary embolism on CT angiography [43].

### 3.2. Endoscopy

#### 3.2.1. Detection of Lesions and Neoplasm

The prognosis of gastric cancer depends heavily on the stage at diagnosis, with 5-year survival rates ranging from over 90% for pathological stage IA to 16.4% for stage IV [44]. Early endoscopic detection of gastric cancer not only reduces mortality but also facilitates organ-preserving treatments, such as endoscopic submucosal dissection (ESD), which is associated with lower late complication rates and shorter hospital stays compared to surgery [45]. However, current endoscopic practices face challenges, with 8.3% to 11.3% of upper gastrointestinal (UGI) cancers missed within 3 years before diagnosis [46,47,48]. UGI endoscopy also shows significant operator dependence, affecting diagnostic accuracy [49,50].

An intelligent quality-control system (IQCS) utilizing CNN was employed to compare the quality of esophagogastroduodenoscopy (EGD) with and without IQCS assistance. The detection rate of early UGI neoplasms in the IQCS group (6.1%) was significantly higher than in the routine group (2.3%). Furthermore, the early UGI detection rate improved in both non-academic and academic centers, as well as for both less-experienced and experienced endoscopists. This suggests that the IQCS can enhance diagnostic accuracy, particularly in primary care hospitals and among non-expert endoscopists [51]. Another model based on the Single Shot MultiBox Detector (SSD) accurately diagnosed 71 out of 77 gastric cancer lesions from 2296 test images, achieving an overall sensitivity of 92.2% in just 47 s. The missed lesions were all superficially depressed and differentiated-type intramucosal cancers, which are difficult to distinguish from gastritis, even for experienced endoscopists [52]. Another model was also able to analyze 2940 images in 45.5 s with 58.4% sensitivity, compared to an endoscopist’s 31.9% sensitivity over 173 min [53]. These AI applications demonstrate potential for enhancing diagnostic accuracy and efficiency in both real-time endoscopy and post-procedure image review, potentially reducing the risk of overlooked cancers.

#### 3.2.2. Determining Depth of Cancer Invasion

Accurately diagnosing the invasion depth of esophageal, gastric, and colon cancers is crucial for appropriate treatment selection. Conventional endoscopy with white light imaging (WLI) is the most widely used and simplest option for diagnosing. It involves evaluating features such as irregular surface, marginal elevation, and converging folds in gastric cancer [54], while in colon cancer, features including tumor size and pit pattern [55] provide key information for determining the depth of invasion. However, because these diagnostic criteria are subjective and unclear, accurately determining invasion depth using WLI requires extensive experience and time [56]. In addition to WLI, various advanced diagnostic techniques, including magnifying endoscopy with narrow band imaging (NBI) and endoscopic ultrasound (EUS), are employed to assess invasion depth more accurately. EUS is highly accurate in distinguishing between early and advanced gastric cancer, but its accuracy in differentiating T1m (mucosal) from T1sm (submucosal) cancer is limited. While EUS shows an overall accuracy of 65–92% for gastric cancer staging, its accuracy decreases to 70–76% when applied specifically to early gastric cancer (EGC). However, since ESD is recommended for patients whose cancer invasion is limited to the mucosa or superficial submucosa, accurately distinguishing these stages is crucial for selecting appropriate candidates for endoscopic resection. Furthermore, EUS is not available in all hospitals and requires additional expense [57].

AI models have shown promising results in addressing these challenges. A Single Shot MultiBox Detector-based model achieved an AUC of 0.7873 for diagnosing the invasion depth of esophageal squamous cell carcinoma, surpassing the performance of 13 board-certified endoscopists, whose AUC was 0.7058. Furthermore, the AI-diagnostic system dramatically reduced the time needed to assess invasion depths, analyzing 279 images in just 0.1 min compared to the average 36.0 min required by endoscopists [56]. A GoogLeNet-based model was used to detect colorectal tumors and assess their invasiveness, achieving an accuracy of 91.1%, sensitivity of 91.2%, and specificity of 91.0% in identifying noninvasive and superficially invasive neoplasms. The model demonstrated higher accuracy compared to EUS, which achieved an accuracy of 79.3% [58]. A ResNet50 architecture-based model was used to determine the invasion depth of gastric cancer, achieving an AUC of 0.94 with a sensitivity of 76.47%, specificity of 95.56%, and an overall accuracy of 89.16%. The system demonstrated significantly higher accuracy (by 17.25%) and specificity (by 32.21%) compared to 17 human endoscopists, including both experienced and less-experienced (junior) practitioners [59].

#### 3.2.3. Reducing Blind Spot Rate

To improve the endoscopic detection of EGC, doctors must follow basic principles, such as avoiding blind spots, which involve mapping the entire stomach. For comprehensive mapping, at least 22 endoscopic photos covering the essential areas of the stomach are recommended [60]. The British Society of Gastroenterology and the Association of Upper Gastrointestinal Surgeons of Great Britain and Ireland also strongly recommend that a complete EGD should assess all relevant anatomical landmarks and high-risk stations as a minimum standard. These guidelines emphasize the importance of mapping the entire stomach, ensuring a thorough and systematic examination of the upper gastrointestinal tract to avoid blind spots [61]. However, these protocols are often not consistently followed, and endoscopists may overlook certain areas of the stomach due to subjective factors or limitations in their technique. As a result, well-known blind spots can be missed, including the cardia, greater curvature of the upper part, posterior area of the body, pyloric area, and lesser curvature of the antrum [62].

A GoogLeNet-based CNN was trained to classify anatomical locations in EGD images into four major regions (larynx, esophagus, stomach, and duodenum), with additional sub-classifications for stomach images (upper, middle, and lower regions). ROC curve analysis demonstrated the high performance of the trained CNN in classifying these anatomical locations, with AUCs of 1.00 for the larynx and esophagus, 0.99 for the stomach and duodenum, and 0.99 for the specific sub-classifications within the stomach [63]. VGG-16 and ResNet architectures were used to train a program capable of classifying EGD images into 10 stomach parts with an accuracy of 90.2%, and into 26 sites with an accuracy of 63.8%, along with perfect intraobserver agreement (κ = 1.0). The system demonstrated accuracy similar to that of experts and superior to seniors and novices. Additionally, the system could automatically capture images during endoscopy and map them onto a grid model of the stomach, alerting the operator about potential blind spots. This feature provides real-time assistance, ensuring that the entire stomach is thoroughly observed during the procedure [64]. Beyond real-time use, AI can classify 20 images in just 5.2 s, enabling the classification of images from 100 patients in less than 9 min for an EGD procedure, thus allowing efficient differentiation between complete and incomplete tests [65].

In addition to comprehensive mapping, bowel preparation is crucial for accurate colonoscopy, as inadequate cleansing can lead to missed lesions, prolonged procedures, and the need for repeat colonoscopies. A standardized bowel-preparation rating scale, such as the Boston Bowel Preparation Scale (BBPS), can enhance the assessment of missed lesions and guide appropriate screening and surveillance intervals [66]. CNNs trained to evaluate cleanliness according to BBPS achieve up to 89.58% accuracy within 5 to 11 s, allowing quick determination of preparation adequacy [67].

#### 3.2.4. Diagnostic Challenges of Misclassification in AI-Assisted Endoscopy

One of the most critical outcome measures in both automated detection and characterization of neoplasia is the occurrence of false-negative findings—cases where neoplastic lesions are misdiagnosed as non-neoplastic. Such errors can have serious clinical implications, including delayed diagnosis, progression to advanced disease stages, and reduced chances of curative treatment. As previously noted, a deep learning model failed to detect 6 out of 77 lesions, most of which were superficially depressed and difficult to identify, even for experienced endoscopists [52]. Similarly, a model based on the VGG-16 architecture demonstrated an overall AUC of 0.851 for T-staging of early gastric cancer (EGC). However, the model’s accuracy was significantly affected by the histopathologic differentiation of the tumor. In particular, undifferentiated-type histology—especially in T1b lesions—was associated with incorrect depth assessment by the AI system. This observation mirrors the limitations seen in EUS, which also exhibits low accuracy in evaluating invasion depth in T1b tumors [68]. These findings underscore the need for further refinement of AI systems, especially in complex scenarios such as undifferentiated-type EGC and T1b-stage lesions, where diagnostic precision is crucial for guiding appropriate treatment decisions. A key contributor to false-negative results may be the limited quantity and diversity of training data. Enhancing datasets by incorporating a broader range of endoscopic images—particularly dynamic, video-based imagery as opposed to static images—could improve diagnostic accuracy [69].

### 3.3. Pathology

#### 3.3.1. Automated Detection of Out-of-Focus Areas

The slide digitization process using whole-slide imaging (WSI) scanners can create out-of-focus (OOF) areas, despite auto-focus mechanisms. These OOF regions can hinder accurate diagnoses and are difficult to detect, especially when localized. Rescanning after pathologists have spent significant time reviewing other areas can cause workflow delays, and manual review of OOF regions is inefficient. To address this challenge, ConvFocus, an automated focus quality detection system, can identify and classify OOF regions as small as 32 µm × 32 µm in gigapixel-sized WSIs. ConvFocus showed broad applicability across various stain types, tissue types, and scanners, achieving Spearman rank correlations of 0.81 and 0.94 on two different scanners when compared to pathologist-graded focus quality [70]. Similarly, DeepFocus achieved an average accuracy of 93.2%, demonstrating the potential of AI to reduce the impact of focus-related issues on clinical workflows [71].

#### 3.3.2. Automated Cell Quantification

In invasive breast cancer, mitotic counting of the region with the highest proliferative activity, known as the hotspot, is essential for histological grading. However, variability arises in the selection of hotspots and mitotic counting due to subtle differences between mitotic and normal cells. A CNN can preselect a $2\;{\mathrm{mm}}^{2}$ circular hotspot in H&E-stained sections. This hotspot is then provided to pathologists for mitotic count assessment. Baseline interobserver agreement for glass slide assessment without the CNN showed κ = 0.689, whereas using the CNN-generated hotspot in WSI increased the agreement score to 0.814. Additionally, the model’s mitotic counting showed an average κ = 0.724 compared to human observers [72]. Another CNN with max-pooling layers, operating on raw RGB data without human input, generates a mitosis probability map for each pixel, which is then smoothed and processed using non-maxima suppression to identify mitosis centers, achieving an F-score of 0.782 [73].

AI can also quantify tumor-infiltrating lymphocytes (TILs) in H&E-stained sections, which is typically manually evaluated and reported as the proportion of immune cell infiltration in the stromal and tumor compartments. To guide the annotation of training sets, pan-leukocyte CD45 antibody staining was used to identify leukocyte-rich and leukocyte-poor regions. After segmenting the images into superpixels, a CNN was employed to extract features from these superpixels, which were then classified using a support vector machine. The model achieved an F-score of 0.94 in distinguishing immune cell-rich and immune cell-poor regions across 123,442 superpixels. The mean agreement between the automated quantification and assessments by two pathologists was 90% (κ = 0.79), consistent with the inter-observer agreement between the pathologists (90%, κ = 0.78) [74].

Paneth cell (PC) dysfunction and loss are established risk factors for diseases of the small intestine, including Crohn’s disease (CD). Recent advancements in two-stage U-net deep learning have yielded efficient and accurate methods for quantifying PC density from H&E-stained slides, showing strong correlation with expert-validated data. In a validation cohort, this method confirmed that lower PC density in CD patients correlates with a higher risk of recurrence. Specifically, patients in the lowest quartile of PC density experienced significantly shorter recurrence-free intervals. This highlights PC density’s potential as a clinical biomarker for CD prognosis, while offering improved speed and reduced mental fatigue compared to manual quantification in clinical practice [75].

#### 3.3.3. Linking Morphological Features to Molecular and Genomic Profiles

Decision-making in oncology is becoming increasingly complex due to the expanding landscape of predictive and prognostic molecular biomarkers, which facilitate personalized cancer treatment. DNA mismatch repair deficiency (dMMR) testing exemplifies this trend, being crucial for diagnosing Lynch syndrome and identifying microsatellite unstable (MSI) tumors eligible for immunotherapy. While gastrointestinal cancer patients typically have limited benefits from immunotherapy, those with MSI tumors show significant responsiveness to immune checkpoint inhibitors. PCR-based microsatellite testing is the standard technique, alongside other methods like next-generation sequencing and immunohistochemistry (IHC). A combined approach using IHC and a molecular MSI test is recommended for optimal detection [76]. However, many patients outside tertiary care centers remain untested due to accessibility and cost constraints, highlighting the need for affordable MSI testing tools to broaden access to immunotherapy. H&E histology, being widely available and cost-effective, offers a reproducible method to predict MSI status directly or as an initial screening tool by identifying key features such as tumor-infiltrating lymphocytes [77].

Deep learning models offer potential solutions for efficient, universal MSI screening using H&E histology. MSINet, a model based on a modified MobileNetV2 architecture, analyzes WSIs by dividing them into tiles, computing MSI probabilities for each tile, and aggregating these probabilities to determine patient-level MSI status using a predefined threshold. The MSINet model achieved an AUROC of 0.779 on the external dataset, an NPV of 93.7%, a sensitivity of 76.0%, and a specificity of 66.6%. In the reader experiment with 40 cases, MSINet outperformed human experts, achieving an AUROC of 0.865 compared to the mean AUROC of 0.605 for five pathologists. It could potentially reduce the number of patients requiring confirmatory testing by 62.8% while maintaining a high negative predictive value, thus offering significant labor and cost savings in universal MSI testing [78]. Another CNN model based on the ResNet18 architecture, trained on colorectal cancer samples from The Cancer Genome Atlas (TCGA), achieved a patient-level AUC of 0.84 on an external validation set. However, when trained on gastric adenocarcinoma samples from TCGA and tested on gastric cancer samples from the Asian KCCH cohort—which has a very different histology from non-Asian populations—the model’s AUC dropped to 0.69. Additionally, a model trained on colorectal cancer samples performed better than a model trained on gastric adenocarcinoma when applied to colorectal cancer samples. This performance discrepancy highlights a limitation in the model’s ability to generalize beyond the cancer type and ethnicity represented in the training set, emphasizing the need for larger, more diverse training cohorts that include rare morphological variants [79].

Predictive modeling of mutational status also plays a critical role in guiding targeted therapy. An Inception-v3 architecture-based CNN trained on WSIs accurately classified tissue types into adenocarcinoma, squamous cell carcinoma, or normal lung tissue with an AUC of 0.97, comparable to pathologists’ performance. Furthermore, it predicted mutations in key genes such as STK11, EGFR, FAT1, SETPB1, KRAS, and TP53 directly from pathology images, which has significant implications for tailoring therapies such as tyrosine kinase inhibitors for patients with EGFR mutations [80]. Another Inception v3-based model demonstrated 96% accuracy in classifying benign versus malignant tumors in hepatocellular carcinoma, matching the performance of an experienced pathologist. This model also successfully predicted mutations in genes like CTNNB1, FMN2, TP53, and ZFX4 from histopathology images, achieving external validation AUCs of 0.71 to 0.89 [81]. While many proof-of-concept studies for deep learning in mutation prediction have reported AUROC values in the range of 0.70–0.90 (corresponding to a specificity of 50% at 90–95% sensitivity), which falls below the threshold for a definitive diagnostic test, such models could still be valuable for pre-screening patients for rare mutations, reducing the burden of molecular testing required [82].

#### 3.3.4. Prognosis Prediction

In oncology, the risk of relapse or death is a pivotal factor in therapeutic decision-making. For instance, high-risk stage II or III colorectal cancer patients may be considered for adjuvant chemotherapy post-surgery, while high-risk stage IV CRC patients might be recommended more aggressive systemic therapies than those currently recommended by clinical guidelines [82]. Traditionally, the tumor–node–metastasis (TNM) system and histologic subtype have been the basis for prognostication and treatment planning. However, additional histologic features have shown prognostic value, such as stromal CD8+ TILs in non-small cell lung cancer [83], heterogeneous chromatin organization in multiple solid tumors (e.g., colorectal and ovarian cancers) [84], and nuclear morphometric features in breast cancer [85].

Deep learning approaches have demonstrated significant potential in improving prognostication. A combined convolutional and recurrent neural network model successfully predicted the five-year outcome of colorectal cancer using a single H&E-stained tumor tissue microarray (TMA) spot image per patient. This deep learning-based approach outperformed visual histological assessments by human experts (hazard ratio [HR] 2.3 vs. HR 1.67) in stratifying patients into low- and high-risk categories [86]. Another CNN calculated the “deep stroma score”, a prognostic metric derived by combining tissue components with a hazard ratio greater than 1, weighted by their prognostic significance, to assess survival outcomes based on non-tumor (stromal) features in colorectal cancer tissue. In external validation, the deep stroma score was found to be an independent prognostic factor for overall survival (HR 1.63), colorectal cancer-specific overall survival (HR 2.29), and relapse-free survival (HR 1.92) [87].

A CNN model for HER2-positive breast cancer prognosis prediction, where about 30% of patients experience recurrence or metastasis after trastuzumab therapy, achieved an AUC of 0.72 on independent testing data using H&E images and clinical data, despite significant variability in patient demographics and experimental conditions [88]. HCC-SurvNet was developed to predict recurrence-free interval (RFI) risk scores after curative-intent surgical resection for hepatocellular carcinoma (HCC), directly from WSI of H&E-stained tissue samples. Statistically significant differences in survival were observed between low- and high-risk subgroups stratified by the model’s risk scores, outperforming the standard AJCC/UICC staging system in predicting post-surgical HCC recurrence risk. The HCC-SurvNet risk score was shown to be an independent predictor of RFI in both internal (HR 7.44) and external (HR 2.37) test sets [89]. The Clinical Histopathology Imaging Evaluation Foundation (CHIEF) model, using histopathology images from initial diagnoses, effectively distinguished between patients with longer-term and shorter-term survival across seven cancer types. CHIEF achieved an average concordance index (c-index) of 0.74, surpassing the state-of-the-art (SOTA) deep learning models PORPOISE (0.62) and DSMIL (0.67), and demonstrated even greater accuracy in independent cohorts (c-index of 0.67 vs. 0.54 and 0.58), highlighting its robust performance and generalizability [90].

#### 3.3.5. Optimizing AI for Efficiency, Affordability, and User Experience

A major challenge in applying machine learning techniques to image classification is the availability of labeled training data. Whole slide pathology images are large and complex, making it both expensive and time-consuming for experienced human observers to produce the necessary labeled data. As a result, supervised learning, which relies on labeled datasets, faces these difficulties. On the other hand, unsupervised learning, which uses unlabeled datasets, requires less human effort but cannot validate the model’s performance as effectively. Therefore, semi-supervised learning methods, which leverage a small amount of labeled data alongside a larger amount of unlabeled data, have emerged as a promising solution. One such semi-supervised method, based on the Mean Teacher architecture, has been shown to achieve performance comparable to supervised learning with extensive annotations in colorectal cancer recognition, closely matching the AUC of human expert pathologists in diagnostic accuracy. This suggests that semi-supervised learning can significantly reduce the amount of annotated data required for achieving expert-level performance in pathological image analysis [91]. Another semi-supervised model, based on the cluster-then-label technique, relies on the cluster assumption, which suggests that unlabeled data points within the same class are likely to form dense clusters in the data space. By identifying these high-density regions, the model can then use a supervised support vector machine to determine the decision boundary. This approach enhances the learning process by incorporating both labeled and unlabeled data to refine the decision boundary [92]. The Reliable-Unlabeled Semi-Supervised Segmentation (RU3S) model differs from traditional semi-supervised learning methods by evaluating the reliability of unlabeled samples through correlation, allowing the model to prioritize more reliable samples for training. Compared to existing semi-supervised learning models, including the SOTA semi-supervised segmentation model ST, RU3S achieves a 2.0% improvement in mIoU accuracy and proves effective in handling complex cytopathological image segmentation tasks [93].

The Augmented Reality Microscope (ARM) offers a cost-effective way to integrate deep learning into pathology imaging. It overlays AI-generated information onto the sample in real time, aligning with the observer’s field of view and compensating for small changes in eye position. This approach eliminates the need for expensive infrastructure investments to transition from analog microscopes and overcomes difficulties in using external AI algorithms due to hardware and software discrepancies. The ARM system successfully applies pre-existing algorithms, despite being trained on images from different digitization methods. It has demonstrated high accuracy in detecting various types of cancer. Two deep learning models for detecting metastatic breast cancer and prostate cancer achieved AUC scores of 0.92 and 0.93 at ×10 magnification, and 0.97 and 0.99 at ×20 magnification, respectively. Key features of the ARM include the ability to provide tumor region outlines, metastasis size, and Gleason pattern breakdowns, and can also be used for stain quantification, cell counting, and microorganism detection [94].

The integration of natural language capabilities into AI systems marks a significant leap forward in computational pathology. This advancement enhances the accessibility, interpretability, and collaborative potential of AI tools, making them more user-friendly and effective in supporting clinical decision-making processes. Recent advancements have led to the development of multimodal generative systems, such as PathChat, powered by multimodal large language models (MLLMs), capable of processing both visual and textual inputs. These systems are trained on SOTA vision-only encoders for histology images and aligned with textual data using large datasets of image-caption pairs. By integrating visual encoders with advanced language models and fine-tuning for specific tasks, these systems can respond to complex pathology queries. Such multimodal AI systems have demonstrated high performance not only in answering multiple-choice diagnostic questions but also in open-ended queries, especially when both clinical context and images were provided, and even in interactive multi-turn conversations. Notably, PathChat has outperformed other publicly available models like LLaVA and LLaVA-Med, as well as the commercial solution GPT-4V, in terms of diagnostic accuracy and response quality. This superior performance is achieved despite PathChat being a smaller and more cost-effective model to deploy [95].

#### 3.3.6. Clinical Validation of AI-Based Pathology Tool

An AI-based pathology system, AI-based measurement of metabolic dysfunction-associated steatohepatitis (AIM-MASH), was developed to assist pathologists in MASH trial histology scoring. Involving over 1400 biopsies and 13,000 independent reads from four global clinical trials, AI-assisted evaluations demonstrated superior accuracy to manual scoring for inflammation, ballooning, and composite endpoints (MAS ≥ 4 and MASH resolution), while maintaining non-inferiority for steatosis and fibrosis. In repeatability studies, AIM-MASH exceeded a predefined 85% performance benchmark, outperforming manual intra-pathologist agreement (e.g., steatosis, κ = 0.72). Reproducibility across labs was higher than published interpathologist variability among experts (e.g., steatosis, κ = 0.63). Clinically, AIM-MASH consistently aligned individual pathologist reads closer to gold standard consensus reads, particularly for challenging components like hepatocellular ballooning. In drug trials, AIM-MASH results either reproduced or enhanced primary efficacy findings, identifying statistically significant treatment effects that were not detected by manual pathology assessments. These results highlight AIM-MASH’s potential to standardize histologic assessments, reduce reader variability, and enhance the accuracy and reliability of endpoint evaluation in MASH clinical trials [96].

## 4. AI in Treatment

**Figure 2:**
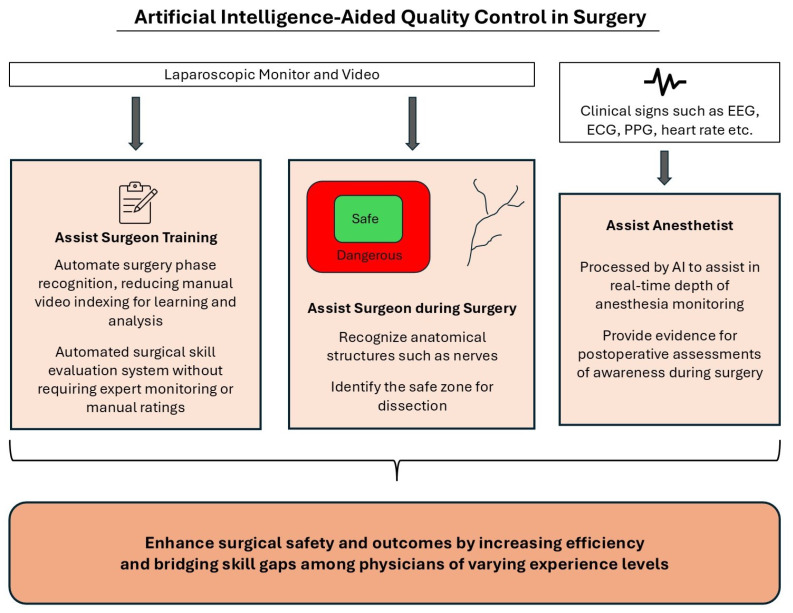
Artificial Intelligence-Aided Quality Control in Surgery.

AI is used in surgery to assist both surgeons and anesthetists, as illustrated in Figure 2. For surgeons, AI supports key areas such as training and surgical procedures, while for anesthetists, it aids in monitoring and decision-making during anesthesia management.

### 4.1. Surgery

#### 4.1.1. Anatomical Structure Recognition

AI-supported recognition systems assist surgeons in identifying anatomical structures, reducing misidentification errors that account for 30% of surgical complications. A U-Net architecture-based model was developed to identify thoracic nerves during lymph node dissection in lung cancer surgery. The system was employed in ten lung cancer surgeries, achieving a Dice coefficient of 0.56 and a Jaccard index of 0.39. The AI monitor exhibited minimal time lag compared to the thoracoscopy monitor and successfully recognized the recurrent laryngeal nerve, as well as the vagus, phrenic, and abdominal nerves. Its accuracy remained consistent across various surgical procedures (thoracic or abdominal), positions, scope rotations, and organ exposure methods [97]. Similarly, a DeepLabv3+-based CNN for detecting the recurrent laryngeal nerve during thoracoscopic esophagectomy achieved an average Dice coefficient of 0.58, comparable to specialized esophageal surgeons and outperforming general gastrointestinal surgeons [98].

AI has also been applied to define safe dissection planes. In gastrectomy, a U-Net-based model for segmenting loose connective tissue fibers achieved a mean Dice coefficient of 0.549. The model received a mean sensitivity score of 3.52 out of 4 in qualitative evaluation, with 88% of evaluations rating the AI segmentation as highly convincing (score 4) [99]. For laparoscopic cholecystectomy, a ResNet50-based model identified safe and dangerous zones with a Dice coefficient exceeding 0.70, and recognized key anatomical structures, including the gallbladder, liver, and hepatocystic triangle. The model outputs were visualized in two formats: a binary format and a topographical heat map. Unlike binary classifications, the heat map provided a pixel-wise probability of each region being part of a zone, offering a more nuanced representation of surgical anatomy. This approach accounts for indeterminate regions, reflecting the complexity of surgical anatomy [100].

In dental implantology, AI aids in mandibular canal segmentation for proper implant positioning. A 3D CNN achieved 0.5 mm prediction accuracy for 90% of the mandibular canal length, meeting the requirements for safe implant placement [101]. A U-Net architecture-based model also demonstrated a high accuracy of 0.998 and a consistency score of 1, surpassing the manual segmentation accuracy of 0.910. Notably, this AI model completed the segmentation process 284 times faster than traditional manual methods using cross-sectional slices in 3D imaging software. It demonstrates not only high accuracy but also exceptional time efficiency in pre-surgical planning [102]. Other studies have also demonstrated the use of U-Net-based models for the automated segmentation in CBCT scans [103,104].

#### 4.1.2. Surgical Workflow Recognition

Modern operating rooms utilize various computer-assisted systems, but these are not always in constant use during surgery. Context-aware systems address this by automatically presenting relevant information based on the current surgical phase, reducing the need for manual navigation of multiple displays. This streamlines operations and provides a tailored user interface for surgeons [105]. In minimally invasive surgery, surgeons often encounter challenges like inaccurate plane dissection and visceral injuries due to unfamiliar views and complex anatomy. While video-based learning can help mitigate these issues, the process of manual indexing of videos is time-consuming. AI with surgery phase recognition capabilities can automate this learning process, enhancing efficiency [106]. These AI systems can analyze surgical procedures using visual data from images or videos, or through multimodal data that incorporates kinematic information from surgical gesture recognition [107].

Recent advancements in AI-based surgical workflow recognition have shown promising results across various procedures. A CNN model trained on 300 laparoscopic colorectal surgery videos achieved high accuracies in classifying surgical phases(81%) and actions (83.2%) [108]. The same research team developed a model for recognizing the transanal total mesorectal excision (taTME) procedure, which is challenging due to infrequent changes in the operative field and instruments. This model achieved 93.2% accuracy in classifying the five major steps of TaTME and 78% accuracy when sub-step classification was included [106]. For ESD workflow recognition, an AI model called AI-Endo was developed to analyze procedures across different organs, including the esophagus, colorectum, and stomach. AI-Endo achieved an average workflow recognition accuracy exceeding 86.66%. To evaluate its real-time computational efficiency and clinical compatibility, both ex vivo and in vivo animal trials were conducted, demonstrating its potential for integration into standard clinical setups [109].

In robotic-assisted partial nephrectomy (RAPN), deep learning techniques (CRNN and sequence models) were employed to recognize surgical steps, while also using an ontology-based Surgical Process Model to capture the meaning and relationships between tools, actions, and steps during the procedure. This approach goes beyond simply recognizing surgical phases by considering the entire sequence of actions and incorporating semantic information. The system successfully identified 10 RAPN steps with a prevalence-weighted macro-average F1 score of 0.76, based on analysis of nine RAPN videos [110].

Additionally, AI systems have been developed to combine visual and kinematic data from robotic surgery. One approach uses interactive message propagation (a technique used to exchange and integrate information between different data types) in the latent feature space. The system extracts features from both video and kinematic data using Temporal Convolutional Networks (TCNs) and Long Short-Term Memory (LSTM) networks. It then identifies the multi-relations between these two data types and utilizes them through a hierarchical relational graph learning module, which captures both simple and complex relationships to enhance the understanding of surgical gestures [111]. Another study employs a multimodal attention mechanism within a two-stream TCN framework. This approach processes visual and kinematic data separately in unimodal streams before combining their outputs. The results obtained on the JIGSAWS (Joint International Group for the Study of Robotic Surgery) benchmark dataset and a new in vivo dataset demonstrate higher accuracy and better temporal structure than unimodal solutions [112].

#### 4.1.3. Surgical Skill Assessment

Surgical training is crucial for patient safety and optimal outcomes, especially in advanced techniques like minimally invasive and robot-assisted surgery. Traditional assessment methods, such as expert monitoring and manual ratings, are becoming inadequate due to their time-consuming nature and potential for bias. Consequently, there is a growing interest in automated surgical skill evaluation systems, which can provide objective, standardized assessments and enable more efficient training even in the absence of human supervisors [113,114].

A model based on 3D ConvNet architecture was able to assess technical skill from video data by analyzing multiple short snippets through a Temporal Segment Network instead of entire videos, forming a consensus to determine the overall video classification and simplifying data processing. Its method achieved classification accuracies exceeding 95% on the JIGSAWS dataset, effectively distinguishing between expert, intermediate, and novice surgeons [114]. Another model achieved accuracies above 91% on the JIGSAWS dataset, successfully interpreting skills within a 1–3 s window without the need to observe the entire training trial [113]. A similar study demonstrated an automatic skill assessment for purse-string suturing in taTME, correlating with manual skill assessment scores, length of suture time, and the surgeon’s experience [115].

#### 4.1.4. Depth of Anesthesia Monitoring

Monitoring brain function, such as using the bispectral index (BIS) processed from electroencephalogram (EEG) to guide anesthesia, can significantly reduce the risk of intraoperative awareness in high-risk surgical patients. Maintaining BIS within the recommended range minimizes anesthetic exposure and decreases the likelihood of postoperative cognitive dysfunction [116,117]. Furthermore, as EEG suppression at low anesthetic concentrations has been associated with an increased risk of postoperative delirium [118], careful monitoring of anesthesia depth during surgical procedures is essential to optimize patient outcomes. However, BIS monitoring is not cost-effective for widespread use due to the high cost of EEG electrodes, the BIS algorithm’s patent restrictions, and the overall cost of the BIS monitor itself [119,120].

Research has focused on directly analyzing EEG signals for real-time depth of anesthesia (DoA) monitoring to assist anesthesiologists in decision-making. One approach extracts four key features from EEG signals. By applying an adaptive neurofuzzy classification algorithm (ANFIS-LH) to these features, the system can accurately classify the data into four different anesthesia states. This method achieved 92% accuracy with sevoflurane anesthesia and 93% accuracy with propofol and volatile anesthesia [121]. Another system, AnesNET, uses a novel EEG-based index called EEGMAC to predict DoA for both inhalational and intravenous anesthetics. Implemented on a cost-effective, compact platform (Raspberry Pi 3), the system delivers real-time predictions with low error rates (0.048 squared, 0.05 absolute) and operates at 20 ms per estimate, significantly outperforming traditional BIS methods in speed [122]. Furthermore, a strong correlation (0.892) was found between the BIS values and the DoA index predicted by the artificial neural network, demonstrating that AI-based methods closely align with the established BIS measure [123].

Research has explored alternative clinical signs to predict DoA beyond BIS and EEG. Electrocardiogram (ECG) and photoplethysmography (PPG) have been investigated due to their low cost and ease of acquisition. A study using MATLAB R2020a-generated heatmaps of ECG and PPG signals as inputs for CNN models. The best-performing model achieved an accuracy of 86% when combined ECG and PPG heatmaps were used as inputs. This approach demonstrates a cost-effective solution requiring minimal data reconstruction, memory, and timing constraints, making it suitable for resource-limited healthcare settings [120]. Another study used end-tidal carbon dioxide concentration, arterial blood oxygen saturation, systolic and diastolic blood pressure, and heart rate to predict awareness with recall during general anesthesia. The model achieved a sensitivity of 23%, specificity of 98%, and a prediction probability of 0.66. While the model’s low sensitivity makes it unreliable for detecting awareness in individual patients during surgery, when patients report experiencing awareness after surgery, the model’s high specificity could provide strong supporting evidence in postoperative evaluations [124].

## 5. **Healthcare Applications and Devices for Personalized Treatment**

Wearable sensors and implantable devices enable real-time monitoring of physiological and biochemical indicators such as heart rate, electromyography (EMG), and blood oxygen saturation. These data are integrated with machine learning (ML) to objectively assess pain severity, identify patterns, and tailor personalized management strategies, reducing diagnostic inconsistencies inherent in traditional subjective methods. For instance, AI-guided drug delivery systems autonomously adjust medication dosages to minimize side effects and dependency risks, ensuring safer and more effective pain management [125]. Complementing these advancements, AI-driven home enteral nutrition (HEN) systems automate personalized care by analyzing patients’ nutritional status and disease characteristics. Through continuous tracking of biomarkers like weight, BMI, and protein levels, these systems optimize nutrient intake, improve BMI regulation, and reduce anemia rates. Early warning mechanisms further enhance safety by alerting patients to malnutrition risks, enabling timely interventions, and ensuring adherence to therapeutic protocols [126]. The integration of biointegrated and implantable optoelectronic devices expands AI’s role in quality assurance. These innovations enable high-precision continuous monitoring and therapeutic interventions (e.g., cardiac pacing, defibrillation) while minimizing patient discomfort. By combining real-time data analysis with closed-loop feedback systems, such technologies ensure consistent care delivery, reduce human error, and align with ethical standards for patient autonomy and safety [127].

## 6. **Challenges of AI in Healthcare**

**Figure 3:**
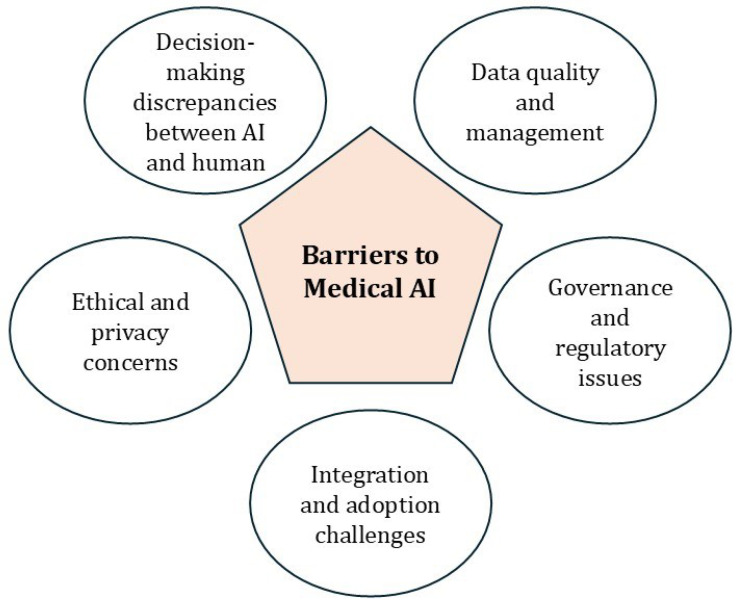
Barriers to medical AI.

The integration of AI into clinical practice presents several challenges that must be addressed to ensure its effective and responsible use in healthcare, as shown in Figure 3. Human decision-making in medicine is context-driven and efficient, allowing for simplification based on relevant cues. In contrast, AI systems process all available information without the ability to ignore extraneous data or question dataset validity. This fundamental difference creates a significant challenge for integrating AI into clinical practice, as AI may use statistically relevant but clinically meaningless features, leading to decisions misaligned with clinicians’ cognitive approaches. This difference presents a major challenge for integrating AI into clinical practice, as AI’s opaque “black box” decision-making hinders trust and complicates clinicians’ ability to predict or understand AI errors and biases [128]. To overcome these challenges, strategies such as developing user-friendly AI interfaces, enhancing clinician trust through Explainable AI (XAI), integrating AI training into medical curricula, and creating protective policies for healthcare professionals are essential [129].

The performance of AI in healthcare also depends heavily on the quality and quantity of input data, with issues like dataset shifts—caused by changes in medical practices, demographics, or emerging diseases—presenting a significant challenge. To ensure robust and reliable AI systems, several strategies are essential: creating accurate, manually annotated reference datasets by experts to standardize algorithm evaluation, validating AI and machine learning tools using multi-institutional data to ensure generalizability, and implementing periodic model retraining and regular output monitoring to mitigate risks and align AI with human values [6,130]. Additionally, the healthcare environment must foster data sharing and collaboration to support continuous AI training [131].

Ethical and safety concerns, including risks of hacking, data breaches, and potential manipulation of algorithms, further complicate AI’s integration into healthcare. Advancements in facial recognition and genomic sequencing technologies further complicate the protection of individual privacy, making it increasingly challenging to maintain anonymity in medical databases [132]. To mitigate these risks, effective governance and regulatory frameworks at the hospital level are critical for ensuring patient safety, ethical compliance, and accountability [129]. As AI continues to evolve and integrate into medical practice, addressing these limitations and challenges will be essential for its successful and responsible adoption.

## 7. Guidelines for AI-Driven Quality Control in the Medical Field

First, it is essential to ensure the quality and standardization of medical data to enhance AI reliability. Medical institutions should use high-quality, well-annotated datasets and follow standardized data processing protocols. Additionally, datasets must be regularly updated and validated to maintain accuracy and relevance in clinical applications [133].

Second, AI should be used to enhance diagnostic accuracy by automating image quality assessment and quality assurance systems. Advanced AI models can detect errors, refine image segmentation, and minimize diagnostic inconsistencies. Implementing real-time feedback mechanisms will further improve the precision of medical imaging interpretation.

Third, AI should play a vital role in surgical guidance and treatment planning. AI-assisted systems can identify anatomical structures with high precision, provide real-time monitoring during procedures, and reduce surgical errors. By integrating AI into personalized treatment planning, healthcare providers can predict patient outcomes more effectively.

Fourth, AI adoption in healthcare must comply with ethical and legal regulations. Strict measures should be taken to protect patient data privacy under laws such as the General Data Protection Regulation (GDPR) and the Health Insurance Portability and Accountability Act (HIPAA) [134]. Additionally, AI should be explainable (XAI) so that medical professionals can understand its decision-making process. Establishing clinical validation procedures is also necessary to ensure AI aligns with standard medical practices.

Fifth, AI models should undergo continuous evaluation and refinement to improve their performance. Regular retraining and validation of AI models with updated datasets will enhance their reliability [135]. Creating a feedback loop with healthcare professionals can further refine AI models and optimize their effectiveness in clinical practice.

Sixth, AI should be integrated as a decision-support tool rather than replacing human expertise. Healthcare professionals should receive adequate training to utilize AI effectively. Additionally, usability studies should be conducted to gather feedback from medical practitioners, ensuring that AI systems remain practical and user-friendly in real-world applications [136].

**Figure 4:**
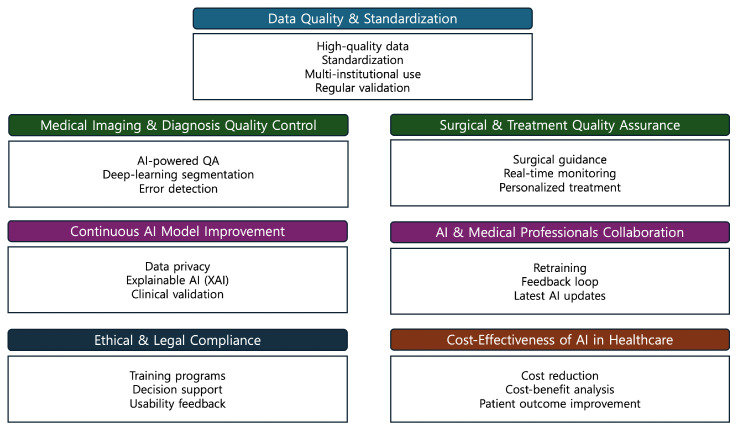
Guidelines for AI-driven quality control in medical diagnosis and surgery.

Seventh, the financial impact of AI implementation in healthcare must be carefully assessed. Hospitals and clinics should conduct cost-benefit analyses to determine whether AI-driven automation reduces operational costs and enhances efficiency [137]. Furthermore, AI adoption should directly contribute to improved patient outcomes and overall healthcare quality. The simplified workflow for the above guidelines is illustrated in Figure 4.

## 8. Conclusions

Artificial intelligence (AI) is rapidly transforming healthcare, offering unprecedented opportunities to enhance diagnostic accuracy, surgical precision, and overall quality assurance. This review underscores AI’s significant contributions across various domains, such as radiology, pathology, and surgery, where advanced neural networks have surpassed traditional methods in tasks ranging from diagnosis and surgical assistance to prognostic modeling. These advancements highlight AI’s ability to process complex data with remarkable speed and precision, reducing diagnostic variability and improving patient outcomes. By automating repetitive tasks and ensuring consistent quality control, AI not only alleviates the burden on healthcare professionals but also sets new standards for efficiency and reliability in medical practice. Furthermore, AI-powered wearable sensors, implantable devices, and personalized nutrition systems demonstrate AI’s transformative potential in delivering tailored treatments through real-time monitoring and dynamic adjustments, enhancing both safety and efficacy in patient care.

However, the integration of AI into healthcare is not without challenges. Issues such as trust between AI and clinicians, ethical concerns surrounding patient privacy, and the limited generalizability of AI models remain significant barriers to widespread adoption. These limitations highlight the need for robust quality control measures to ensure AI systems are both reliable and equitable. This review proposes a set of guidelines aimed at addressing these challenges, including the establishment of standardized data frameworks, rigorous validation protocols across diverse populations, and interdisciplinary collaboration among clinicians, data scientists, and policymakers. These measures are essential for fostering trust in AI technologies while ensuring their safe and effective deployment in clinical settings.

By combining its inherent strengths with these targeted quality control strategies, AI has the potential to overcome its current limitations and further revolutionize medical practice. Standardized frameworks can enhance model generalizability, while ethical governance can address privacy concerns and promote public trust. Together, these efforts will enable AI to not only refine existing applications but also unlock new possibilities in precision medicine. As healthcare continues to evolve, embracing AI’s transformative potential while addressing its challenges through thoughtful guidelines will be key to delivering safer, more personalized care for patients worldwide.

## Figures and Tables

**Table 1:** Types of neural networks.

Terms	Definition
Artificial neural network (ANN)	ANN is a mathematical model that analyzes complex relationships between inputs and outputs. It mimics the human brain by processing various types of data, identifying patterns, and applying them to decision-making tasks [9].
Convolutional neural network (CNN)	CNN is a specialized neural network using convolution structures inspired by the human visual system. It employs local connections, weight sharing, and pooling to reduce complexity, making it particularly effective for image and pattern recognition tasks [10].
Recurrent neural network (RNN)	RNNs process sequential data, such as text, time series, and speech, by using feedback loops to capture temporal dependencies. They are ideal for tasks like speech recognition and video tagging, where understanding the sequence and timing of data is crucial [11].

**Table 2:** Performance metrics used to assess artificial intelligence.

Terms	Definition
Confusion Matrix	A tool used to assess the performance of a classification model by comparing its predicted outcomes with the actual results. For binary classification, the matrix is typically 2 × 2, displaying true positives, true negatives, false positives, and false negatives. In multi-class classification tasks, the matrix is extended to include additional rows and columns to account for all classes, showing the model’s performance in terms of these outcomes for each class [12].
	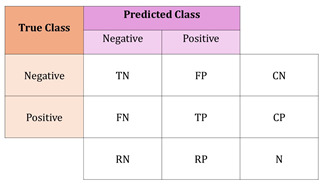
	N = total number of examples
	CN = number of truly negative examples
	CP = number of truly positive examples
	RN = number of predicted negative examples
	RP = number of predicted positive examples
Recall (Sensitivity)	A test’s ability to correctly identify true positives
	Sensitivity = (TP)/(CP)
Specificity	A test’s ability to correctly identify true negatives
	Specificity = (TN)/(CN)
Accuracy	Represents the number of correct predictions over all predictions
	Accuracy = (TN + TP)/(CP + CN) [13]
Precision (positive	Accuracy of positive predictions
predictive value)	Precision = (TP)/(RP)
Negative predictive value	Accuracy of negative predictions
(NPV)	NPV = (TN)/(RN)
F1 score	Harmonic mean of precision and recall
	F1 = 2× (precision × recall)/(precision + recall) [12]
Receiver operating characteristic curve (ROC curve)	A graphical plot that illustrates the performance of a binary classifier system as its discrimination threshold is varied. It is created by plotting the true positive rate (sensitivity) against the false positive rate (1− specificity) at various threshold settings, providing a comprehensive view of the classifier’s performance across different decision thresholds [13].
Area under the ROC Curve (AUROC)	Represents the overall performance of a classification model by assessing its ability to distinguish between positive and negative classes across all decision thresholds. A higher AUC indicates better model discrimination, providing a comprehensive evaluation of the system’s performance over varying operating points, rather than relying on a single sensitivity-specificity pair [13].
Mean Absolute Error (MAE)	Average absolute differences between predicted and actual values.
Mean Squared Error (MSE)	Average squared differences between predicted and actual values, emphasizing larger errors [14].

## Data Availability

The data that are discussed in this article are presented in cited studies.
